# Predicting Overall Survival for Patients with Malignant Mesothelioma Following Radiotherapy via Interpretable Machine Learning

**DOI:** 10.3390/cancers15153916

**Published:** 2023-08-01

**Authors:** Zitian Wang, Vincent R. Li, Fang-I Chu, Victoria Yu, Alan Lee, Daniel Low, Drew Moghanaki, Percy Lee, X. Sharon Qi

**Affiliations:** 1 Department of Radiation Oncology, University of California Los Angeles, Los Angeles, CA 90095, USA; 2 Department of Biology, University of Southern California Dornsife School of Arts and Sciences, Los Angeles, CA 90089, USA; 3 Department of Medical Physics, Memorial Sloan Kettering Cancer Center, New York, NY 10065, USA; 4 Department of Radiation Oncology, City of Hope Orange County Lennar Foundation Cancer Center, Irvine, CA 92618, USA

**Keywords:** mesothelioma, radiation therapy, overall survival, interpretable machine learning, outcome prediction and assessment

## Abstract

**Simple Summary:**

Malignant pleural mesothelioma (MPM) is a rare cancer arising from the cells of the thoracic pleura of the lungs. It has a poor prognosis and is often fatal, with a reported 5-year survival rate of less than 5%. Post-surgery radiotherapy is recommended for patients with resectable disease to improve local control. However, radiation treatment planning for MPM is extremely challenging due to the anatomic complexity, large surface area, unique concave shape, location, and large size of the pleura planning target volume. The aims of this work are: (1) To examine the achieved dosimetric endpoints for a retrospective cohort of MPM patients and to assess associations of these variables with corresponding overall survival (OS) using machine learning methods; (2) To identify key predictors that influence OS via interpretable machine learning algorithms for left- and right-sided mesothelioma patients separately, and to develop and validate predictive models based on identified clinical and dosimetric parameters for predicting OS.

**Abstract:**

Purpose/Objectives: Malignant pleural mesothelioma (MPM) is a rare but aggressive cancer arising from the cells of the thoracic pleura with a poor prognosis. We aimed to develop a model, via interpretable machine learning (ML) methods, predicting overall survival for MPM following radiotherapy based on dosimetric metrics as well as patient characteristics. Materials/Methods: Sixty MPM (37 right, 23 left) patients treated on a Tomotherapy unit between 2013 and 2018 were retrospectively analyzed. All patients received 45 Gy (25 fractions). The multivariable Cox regression (Cox PH) model and Survival Support Vector Machine (sSVM) were applied to build predictive models of overall survival (OS) based on clinical, dosimetric, and combined variables. Results: Significant differences in dosimetric endpoints for critical structures, i.e., the lung, heart, liver, kidney, and stomach, were observed according to target laterality. The OS was found to be insignificantly different (*p* = 0.18) between MPM patients who tested left- and right-sided, with 1-year OS of 77.3% and 75.0%, respectively. With Cox PH regression, considering dosimetric variables for right-sided patients alone, an increase in PTV_Min, Total_Lung_PTV_Mean, Contra_Lung_Volume, Contra_Lung_V20, Esophagus_Mean, and Heart_Volume had a greater hazard to all-cause death, while an increase in Total_Lung_PTV_V20, Contra_Lung_V5, and Esophagus_Max had a lower hazard to all-cause death. Considering clinical variables alone, males and increases in N stage had greater hazard to all-cause death; considering both clinical and dosimetric variables, increases in N stage, PTV_Mean, PTV_Min, and esophagus_Mean had greater hazard to all-cause death, while increases in T stage and Heart_V30 had lower hazard to all-cause-death. In terms of C-index, the Cox PH model and sSVM performed similarly and fairly well when considering clinical and dosimetric variables independently or jointly. Conclusions: Clinical and dosimetric variables may predict the overall survival of mesothelioma patients, which could guide personalized treatment planning towards a better treatment response. The identified predictors and their impact on survival offered additional value for translational application in clinical practice.

## 1. Introduction

Malignant pleural mesothelioma (MPM) is a rare but aggressive cancer arising from the cells of the thoracic pleura of the lungs. Approximately 3000 new mesothelioma cases are diagnosed each year, accounting for 0.4% of all cancer deaths in the United States [1]. MPM is characterized by its poor prognosis and often fatal nature, with a reported 5-year survival rate of less than 5% [2,3,4]. Mesothelioma is difficult to treat, in part due to its rarity and lengthy latency period. Since it is so rare, it is very likely for mesothelioma to have advanced to a stage that prevents a complete cure before it is diagnosed, as symptoms are minimal.

Macroscopic, complete surgical resection is the cornerstone of management, followed by adjuvant chemotherapy and radiotherapy. However, residual cancer cells are often left behind, as has been demonstrated by the high rate of local post-operative progression [5]. Despite advances in therapy for MPM, the prognosis remains poor, and considerable treatment-associated morbidity is generally expected [6].

Post-surgery radiotherapy (RT) is recommended by the National Cancer Control Network for patients with resectable MPM to improve local control [6]. However, radiation treatment planning for MPM is extremely challenging due to the anatomic complexity, large surface area, unique concave shape, location, and large size of the pleura planning target volume (PTV). The challenges are accentuated by the presence of an intact ipsilateral lung after pleurectomy and decortication, as well as the abutment of multiple critical organs-at-risk (OARs), such as the lungs, esophagus, heart, and spinal cord. As a result, the optimal treatment plan quality may or may not be achieved due to normal tissue safety concerns. Given the potential detriment to survival and/or quality of life whenever target coverage requires overdosing normal structures, we aimed to investigate the associations between patient/target characteristics and achievable plan quality with available clinical endpoints via machine learning (ML) methods.

Artificial intelligence (AI) and Machine learning (ML) methods have demonstrated a wide range of applications and success in building predictive models based on specific datasets for clinical decision-making towards personalized treatment in RT [7,8,9,10]. A convolutional neural network-based classification model, MesoNet, was developed to predict patient survival based on genetic expressions and pathological biopsy features [11]. In a recent study from the European Prospective Investigation into Cancer and nutrition (EPIC) cohort, the authors investigated and identified nine DNA methylation biomarkers in blood preclinical samples. The proposed model shows better predictive value for the identification of high-risk MPM [12]. Traditional ML algorithms, such as support vector machines (SVM), can learn from existing data (with and without clinical labels) and uncover complicated underlying relationships between potential clinical variables and clinical endpoints (such as overall survival, post-RT pneumonitis, etc.). The black box ML methods, however, often do not provide insights into how a predictive model was created, which hinders clinical adoption of such predictive models. Model interpretability, specifically how predictive models make decisions, is paramount for clinical deployment and successful implementation. As an example, the multivariable Cox Proportional Hazards regression model (Cox PH model) is a well-recognized interpretable model for exploring the relationship between the survival of a patient and potential explanatory variables, such as patient clinical characteristics, dosimetric variables, etc. These analyses enable us to further investigate the specific relationship between how each selected predictor impacts overall patient survival [13]. We expect the explainable nature of predictive models to reveal the descriptive impacts of the selected predictors on the clinical endpoints, which allows us to gain a better understanding of the identified predictors and their influences on a specific clinical endpoint, enabling a rapid translation to clinical implementation. Considering that Cox PH regression and survival SVM (sSVM) are two of the most commonly used approaches to evaluating survival data, we opt to focus on assessing both methods for the purpose of validating each other.

The aims of this work are twofold: (1) To examine the achieved dosimetric endpoints for a retrospective cohort consisting of both left- and right-sided MPM patients (LSM and RSM, respectively), and to assess associations of these variables with corresponding overall survival (OS) using machine learning methods; (2) To identify key predictors that influence OS via interpretable ML algorithms for LSM and RSM separately, and to develop and validate predictive models based on identified clinical and dosimetric parameters for predicting OS. According to the laterality of the target, it is expected that for the relevant OARs (i.e., heart, stomach, and liver), their geometric relationships to the target as well as dosimetric endpoints for the OARs could be substantially different between LSM and RSM. We therefore split and performed separate analyses for LSM and RSM in the study. Additionally, we assessed whether the dosimetric differences may translate to clinical endpoints for OARs; to this end, the Normal Tissue Complication Probability (NTCP) for critical structures, such as the heart, lung, and stomach, was estimated. Our hypothesis is that post-RT pneumonitis as well as complications of other OARs may impact OS in MPM patients. Ultimately, our goal is to create an explainable predictive model to facilitate clinical decision making for a personalized, optimal treatment strategy for MPM RT.

## 2. Methods and Materials

### 2.1. Patient Characteristics

We retrospectively analyzed a cohort of 60 MPM patients treated at the Department of Radiation Oncology, University of California, Los Angeles (UCLA), between 2013 and 2018. All patients underwent post-operative radiation therapy following a pleurectomy/decortication. Among all, 37 patients were treated for mesothelioma on the right side (an average age of 69.3 ± 7.9 years old), and 23 patients were treated on the left side (an average age of 66.0 ± 10.4 years old). In terms of histological subtypes, 35 (58.3%) patients were diagnosed with epithelioid MPM, and the remaining patients had biphasic or mixed MPM. The detailed patient demographic and clinical characteristicsare tabulated in Table 1.

### 2.2. Radiation Treatment Planning and Delivery

**Figure 1:**
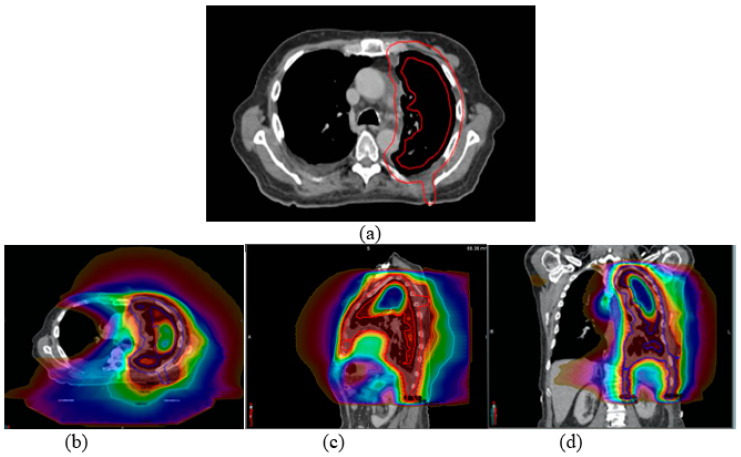
(**a**) The PTV outlined in a transverse view for a sample mesothelioma patient. Sample dose distribution in (**b**) transverse, (**c**) sagittal, and (**d**) coronal views is planned with helical IMRT on a Tomotherapy unit.

All patients underwent post-operative radiation therapy on a Tomotherapy unit (Accuray, Inc., Sunnyvale, CA, USA) using helical intensity modulated radiation therapy (IMRT) with a prescription dose of 45 Gy in 25 fractions. The planning target volume (PTV) was created by expanding the ipsilateral lung contour by 1 cm, and cropping it out of lungs with a margin of 3–5 mm. In the longitudinal direction, the PTV routinely included the surgical scar, drain sites, and any involved nodal regions. Figure 1a illustrates the PTV outlined in a transverse view for a sample MPM patient. The treatment plans were designed with a binary multi-leaf collimator (MLC), a jaw size of 2.5 cm for all cases, and an averaged pitch of 0.33 (0.287, 0.49) with directional blocking of the contralateral lung. Our institution’s dosimetric goals were: PTV V100% (percentage of PTV receiving 100% of the prescription dose) ≥ 95%, spinal cord maximum < 45 Gy, total lung V20Gy < 30%, total lung mean dose < 18 Gy, ipsilateral lung V20Gy < 60%, contralateral lung V5Gy < 70%, heart V22.5Gy < 50%, esophagus mean dose < 24 Gy, and stomach V45Gy < 50 cm^3^ (or 5%). Figure 1b–d shows sample dose distributions in (b) transverse, (c) sagittal, and (d) coronal views planned with helical IMRT on a Tomotherapy unit for the sample case. Each patient was immobilized with a Vac-Loc immobilization device for CT simulation and each treatment delivery. A pre-treatment Megavoltage CT (MVCT) scan was acquired to ensure proper patient alignment before each treatment fraction. Patient-specific volumetric parameters for relevant targets and OARs were contoured on the planning CT. The dosimetric endpoints, such as ipsilateral-, contralateral-, and total lung V20Gy and V5Gy, were collected. The patient-specific volumetric parameters, as well as the achieved dosimetric parameters, were assessed for left-sided and right-sided MPM cases separately. The achieved dosimetric quantities for other OARs, such as the heart, spinal cord, kidneys, and stomach for LSM and RSM, were also analyzed retrospectively.

### 2.3. Statistical Analysis

Descriptive statistics were reported for all metrics, and a Mann–Whitney U-test was performed to evaluate whether the distributional difference of these metrics between the LSM and RSM cohorts exists or not.

### 2.4. Patient Overall Survival (OS) and Survival Prediction

Post-RT patient survival information was obtained from the electronic medical record (EMR) at our institution, which has been in use since 2013. Among the cohort of 60 patients, one patient was excluded from model building due to an unknown diagnosis and treatment date (prior RT at another institution). For patients with records of death dates, the survival time between the initial diagnosis of MPM and the death date was calculated. For patients without a record of death (right-censored patients), the time interval between the initial diagnosis of MPM and the last visit date was calculated. The survival data for each patient contains two elements: the first boolean element indicates whether the event of death happens for the patient, and the second element represents the calculated survival interval. The primary endpoint, overall survival (OS), which corresponds with time from diagnosis to death as a time-to-event variable, constructed in the aforementioned manner, will be estimated by subgroups via the Kaplan–Meier (KM) method, and the difference in OS between subgroups will be assessed via the log-rank test. As the common approach to univariate analysis in survival analysis, the KM method, along with the log-rank test, assesses the survival distribution of the interested endpoint between two groups without accounting for the potential impact of other predictors.

### 2.5. Machine Learning Methods for Predicting Overall Survival

With the approach of multivariable analysis (MVA), ML models, including the multivariable Cox Proporational Hazards (Cox PH) regression model [13] and the survival Support Vector Machine (sSVM) [14], were used to explore the relationship between the OS of a patient and potential explanatory variables and to build predictive OS models from relevant variables. The proportional hazard assumption was examined via the diagnostic plot method. The Cox PH model is a linear regression model specifically for analyzing the impact of clinical variables on OS, while the sSVM is an extension of the standard SVM to right-censored time-to-event data to account for complex non-linear relationships between potential clinical predictors and OS [13,14]. The sSVM treats the survival prediction as a ranking problem based on the estimated risk score.

Clinical variables were taken into ML algorithms to predict OS. The clinical variables were categorized into patient characteristics, including target laterality, age, TN staging (M staging was not included due to limited data to represent the M0 stage in LSM and M stage in RSM), gender, pneumonitis status, histological subtype, and 26 potential dosimetric variables from patient-specific treatment dosimetric plans, e.g., PTV volume, volumes of lung and critical OARs, pre-selected dose-volume metrics, such as mean dose, percent volume receiving X prescription dose (V_X_) for lung, and heart (Supplementary Table S1: full list of potential predictors). For simplicity, TN staging was considered a continuous variable in our analysis. A recursive feature elimination approach, as a backward selection approach via stepwise method (see Supplementary Materials for detail) [15], was applied to select the important features that the final model with clinical variables and dosimetric variables by target laterality, independently and jointly, may reach. The correlation matrix of the selected features to include in the final model, respectively, was obtained.

The performance of the obtained model was validated via k-fold cross-validation, with k being 5 as a rule of thumb. For k-fold cross-validation, the original training cohort was equally split into five subgroups until each sub-group was tested. The performance measure, Harrell’s C-index [16], as the predominated metric to assess the predictive performance for survival analysis, was recorded for each iteration, and the average of the measure was reported to evaluate the fitted model. A C-index of 0.5 means the model randomly predicts the survival ranking, and a C-index of 1 indicates the model correctly predicts all the survival rankings. An estimated ranking based on the predicted risk score will be compared with the real ranking of the survival data set by C-index, which will measure the ranking performance of each model based on the following criteria: (1) For two deceased patients, the patient who lives longer should have a lower risk score. (2) For a deceased patient and a right-censored patient, if the right-censored patient has a longer survival time, the right-censored patient should have a lower risk score.

The level of significance was set to be 0.05. All analyses were carried out via R v4.1.2 [17] with packages *ggplot2* [18], *survival* [19,20], *survminer* [21], *mlr3* [22], *mlr3learners* [23], *mlr3extralearners* [24], *mlr3proba* [25], *mlr3fselect* [26], *mlr3tuning* [27], *paradox* [28], *caret* [29].

### 2.6. Normal Tissue Complication Probability (NTCP)

The potential complication rates, based on achievable treatment planning parameters, for critical OARs were estimated using the Lyman–Kutcher–Burman (LKB) model using the RADBIOMOD program [30]. The most relevant OARs are right- and left-lung, spinal cord, esophagus, heart, liver, left kidney, right kidney, and stomach, as well as their corresponding radiobiology parameters according to Burman et al [31].

## 3. Results

### Achieved Dosimetric Endpoints and Overall Survival

The achieved dosimetric endpoints for target and the selected OARs stratified by target laterality are tabulated in Table 2, and box plots are included in Figure S1. The variables that were statistically different between the LSM and RSM patients are listed in bold numbers. No difference in OS between LSM and RSM patients was identified (*p* = 0.18 by log-rank test), with 1-year OS of 77.3% and 75.0%, respectively. Supplementary Figure S1 shows boxplots of selected dosimetric parameters for critical OARs between LSM and RSM groups. Among these parameters, there were no significant differences between PTV V100 and PTV volume. The ipsilateral lung mean dose and V20Gy, the ipsilateral lung-PTV mean dose and V20Gy, the heart mean dose, and V30Gy, the stomach mean dose, are higher for LSM than those of RSM (*p* < 0.05). The ipsilateral lung-PTV volume, esophagus max dose, contralateral kidney mean dose, liver mean dose, and V30Gy are higher for RSM than those of LSM (*p* < 0.05).

The estimated NTCP for the stomach was higher for LSM than for RSM (*p* < 0.001). There were no statistical differences in the estimated NTCP for total lung, spinal cord, and esophagus between LSM and RSM. The heart, liver, and left and right kidneys each have minimal doses, resulting in an approximated NTCP of 0.

**Figure 2:**
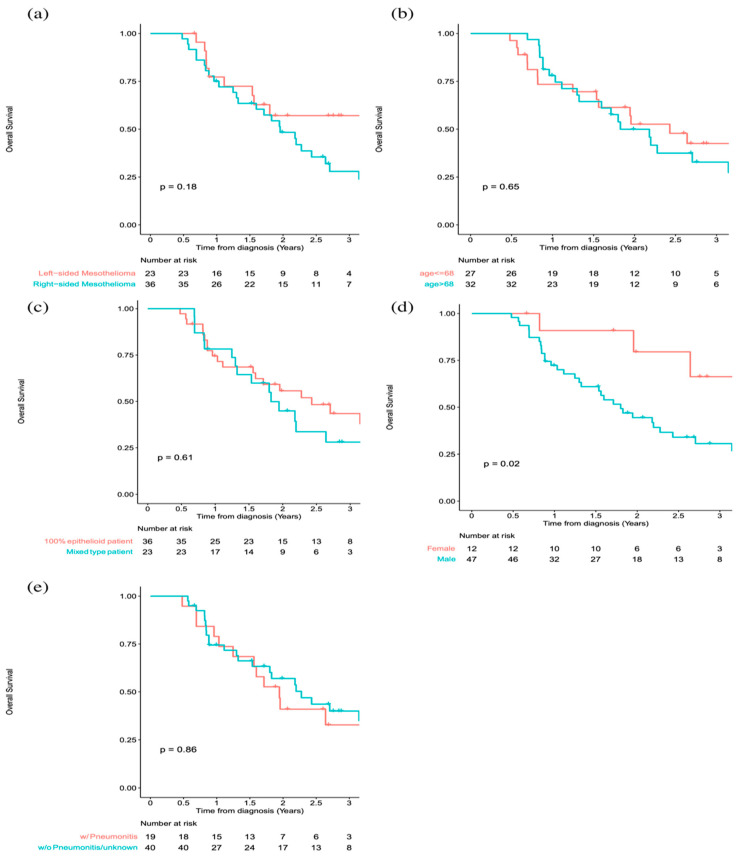
Kaplan–Meier survival curves when patients were stratified according to (**a**) target laterality, (**b**) age, (**c**) histology, (**d**) sexuality, and (**e**) patients with and without pneumonitis in the cohort of MPM patients. A difference (*p* = 0.02) in OS was found between male and female MPM patients as the result of univariate analysis. The mean age of the patient cohort is 68 years old, which is selected to be the cut-off for stratification in (**b**).

Figure 2 illustrates Kaplan–Meier survival curves when patients were stratified according to (a) target laterality; (b) age; (c) histology; (d) sexuality of the MPM patients; and (e) post-RT radiation pneumonitis. No significant difference in OS for each of these categories, including pneumonitis, was found except sexuality.

For ML models based on clinical and dosimetric variables, independently or jointly, the Cox multivariable regression model achieved similar performance as the sSVM model. Specifically, with clinical variables, the Cox PH and sSVM models achieved c-indexes in 5-fold cross validation of 0.70 and 0.65, respectively (Supplementary Table S2).

With Cox PH regression, the model considers dosimetric variables alone on the right side. An increase in PTV_Min, Total Lung PTV Mean, Contra Lung Volume, Contra_Lung_V20, Esophagus_Mean, and Heart_Volume had a greater hazard to all-cause death, while an increase in Total_Lung PTV_V20, Contra_Lung_V5, and Esophagus_Max had a lower hazard to all-cause death (Table 3) and no stable fit may be attained for the model considering dosimetric variables on the left side, likely due to the insufficient information in the limited data for this sub-cohort; for the model considering clinical variables alone, male and increase in N stage had greater hazard to all-cause death (Table 3); for the model considering both clinical and dosimetric variables, increase in N stage, PTV_Mean, PTV_Min, and esophagus_Mean had greater hazard to all-cause death, while increase in T stage and Heart_V30 had lower hazard to all-cause death (Table 3). On the other hand, histology, gender, Pneumonitis, age, side, PTV_max, Total_Lung_PTV_Volume, Ipsi_Lung_PTV_Mean, Esophagus_Volume, Esophagus_Max, Heart_Volume, and Spinal Cord Volume were not found to be predictors that affected all-cause death significantly.

## 4. Discussion

Malignant pleural mesothelioma is an extremely rare but aggressive and fatal cancer. A previous study demonstrated that helical IMRT, compared with conventional 3D-conformal RT, was associated with improved target coverage, which translated into improved local control for MPM [32]. It is known that IMRT optimization is an inverse planning process that generally requires IMRT planning guidelines via trial-and-error approaches because the dosimetric plan quality varies with patient-specific anatomy, planners’ experience, and many other factors. To our knowledge, the planning guidelines for MPM are not well established according to target laterality. We thus performed this study using a relatively large retrospective MPM patient population. Due to its complex concave shape and unique location and geometry (the PTV wraps around and abuts the ipsilateral lung), optimal dosimetry may not always be achieved for MPM cases. For example, general lung constraints of V20Gy ≤ 35% are often not met for MPM plans. Additionally, significant differences in achievable dosimetric endpoints for critical organs were observed given target laterality. Specifically, higher mean doses are expected for the heart and stomach for left-sided cases, while higher liver mean doses are expected for right-sided MPM cases. Given different target laterality, different dose constraints of these normal structures need to be considered during IMRT plan optimization and generation. Additionally, we may need to further reduce doses for certain OARs for patients who have certain risks.

A comparison of OS did not reveal any statistical differences between the LSM and RSM subgroups, nor did age, histology subtypes, or post-RT radiation pneumonitis status. However, a difference in OS was found between male and female patients (*p* = 0.02) as a result of univariate analysis. A similar finding has been reported by Taioli et al. based on a cohort of 14,228 MPM patients from 1973 to 2009: longer survival results were found for female MPM patients [33]. As suggested by Wolf et al., the interaction between high levels of circulating estrogen and estrogen receptors on the tumor may provide protective effects for female MPM patients, which resulted in a longer survival time [34]. As univariate analysis does not account for the impact of other predictors, multivariable analysis, with adjustment for other predictors, is needed to address the research question appropriately. As a result of multivariable analysis with Cox PH regression with dosimetric and clinical variables (Table 3), this gender difference went away, likely because the impact of other predictors on survival has been taken into account.

It is commonly known that there are three distinct histologic subtypes of MPM: epithelioid MPM, sarcomatoid MPM, and biphasic or mixed MPM [35]. Epithelioid MPM is the most common subtype and is associated with the most favorable prognosis [36]. The sarcomatoid subtype has been associated with a worse prognosis and can mimic sarcomas in appearance and behavior [37]. In the current patient cohort, 35/60 (58.3%) of the patients were diagnosed to have epithelioid-type histology, which is generally associated with better outcomes [38,39,40]. There was no patient diagnosed with sarcomatoid subtype histology in the population. The remaining patients had a mixed subtype consisting of epithelioid and sarcomatoid components ranging from 1% to 99%. This study indicated no difference in OS (*p* = 0.61) between the epithelioid and mixed subtypes of mesothelioma cases. A larger sample size is likely needed to further validate the finding.

A previous publication by Allen et al. [41], based on a small cohort of thirteen mesothelioma patients, reported fatal pneumonitis resulting from the sliding window IMRT technique in 6/13 (46.2%) patients. The median time from completion of RT to the onset of radiation pneumonitis was 30 days (range 5–57 days); 4/13 (30.8%) patients developed acute Grade 3 nausea and vomiting [41]. In the current study, we searched our Electronic Health Record (EHR) system for RT-related pneumonitis. Among 60 patients in this study, 14 patients (23.3%) were reported to have definitive radiation pneumonitis after IMRT: 2 patients with grade 2, 1 patient with grade 3, and 11 patients with an unknown level of pneumonitis. Additionally, four patients were reported with possible radiation pneumonitis. The remaining 42 patients reported no or unknown pneumonitis in the EHR. The occurrence of known pneumonitis is significantly lower than the data reported by Allen et al. (84.6%) [41]. We found that no difference in OS for patients with and without pneumonitis exists (*p* = 0.86).

Interpreting the Cox model involves examining the coefficients for each explanatory variable. A positive regression coefficient of an explanatory variable means that the hazard is higher, and a negative regression coefficient implies a better prognosis for patients with higher values of that variable.

Patient characteristics alone were unlikely to be good predictors (c-index = 0.70) to differentiate patient treatment in terms of OS. We hypothesized that the achieved dosimetric endpoints, in addition to patient characteristics, may also potentially affect patient OS. The predictive models, built on patient characteristics alone and combined parameters, resulted in good predictive performance. It is generally considered a big hurdle to translate a black-box predictive model into actual clinical practice due to its lack of interpretability. This study attempted to shed additional light on potential predictors and their explicit relationships with OS. For example, PTV volume is considered to not be impactful enough to OS compared to other variables selected into our final model for the purpose of estimating its impact (Table 3). Among the selected variables, patients with higher values of N stage (for model with clinical variables alone), PTV_Mean, PTV_Min, and esophagus_Mean (for model with dosimetric and clinical variables combined) most likely result in a prediction of higher risk scores and therefore shorter OS, while higher values of Total _Lung_PTV_V20, Contra_Lung_V5, and Esophagus_Max (for model with dosimetric variables alone) may likely result in lower risk scores and thus longer OS for right-sided patients. It is worthwhile to point out that the presence of other confounders, which we did not include in the current study, may play a role in the interest relation, leading the true relationship between these prognostic factors and OS to be concealed. From a clinical aspect, there are studies reporting preclinical models based on genetic expression, with specific point mutations of tumor suppressor genes *BAP1*, *CDKN2A*, and *NF2*, denoting a higher risk of MPM [42,43]. Other genetic expressions that are common in MPM patients include mutations or alterations to PD-L1, VEGF, WT-1, mesothelin, etc., which were identified as prognostic and predictive biomarkers to inform treatment decisions in malignant MPM patients [44]. Further research may help us gain more perspective based on a larger patient sample.

Potential predictors can be identified and validated for other clinical endpoints, including disease-free survival, progression-free survival, loco-regional disease control, etc. We expect the identified predictors could further differentiate individuals and guide future IMRT treatment planning to yield optimal treatment planning quality and achieve optimal clinical endpoints for mesothelioma patients.

Given that the small scale and clinical data are in use for this study, and the literature [45,46] suggests that a C-index around or larger than 0.6 is considered an acceptable or fair prediction, we consider that the predictive performance of the suggested model is fair to arrive at our conclusion. A larger sample size may be needed to further validate our current findings.

## 5. Conclusions

Significant differences in achievable planning dosimetric endpoints for critical structures including the lung(s), heart, liver, kidney, and stomach were observed for mesothelioma patients according to target laterality. We observed no difference in OS between patients with and without pneumonitis. Patient-specific dosimetric quantities along with clinical variables could predict the overall survival of mesothelioma patients (c-index = 0.62 for Cox PH and 0.64 for sSVM). The identified predictors and their impact on survival, revealed by an explainable ML model, offered additional value for translational application towards personalized radiation treatment.

## Figures and Tables

**Table 1:** Patient characteristics for the breakdown of left-sided (23) and right-sided (37) mesothelioma cases included in the study.

Variables	Left-Sided Mesothelioma (n = 23)	Right-Sided Mesothelioma (n = 37)
	Mean ± SD *	Mean ± SD *
**Age (year)**	65.96 ± 10.4	69.25 ± 7.87
**Gender**		
Male	16 (69.6%)	32 (86.5%)
Female	7 (30.4%)	5 (13.5%)
**Staging**		
T1	1	1
T2	6	5
T3	12	26
T4	4	5
N0	10	13
N1	5	6
N2	8	18
M0	22	37
M1	1	0
**Histologic subtype**		
Epithelioid	13	22
Sarcomatoid	0	0
Mixed histology	10	15
**Surgery type**		
Pleurectomy and decortication	23	36 **

Abbreviation: SD *—Standard deviation. 36 **: 1 right-sided mesothelioma patient has missing record.

**Table 2:** Achieved dosimetric endpoints and overall survival for the cohort of 60 mesothelioma patients treated on a Tomotherapy unit stratified by target laterality.

	LSM * (n = 23)		RSM * (n = 37)		LSM vs. RSM
Dosimetric Variables	Median	IQR **	Median	IQR **	*p*-Value
Target					
PTV volume (cc)	2107	750	2193	574	0.82
V100% (%)	94.3	1.0	94.2	2.0	0.42
Lung					
Ipsi lung mean dose (Gy)	**38.9**	**4.5**	**37.1**	**3.5**	**0.02**
**Ipsi lung V20Gy (%)**	**92.0**	**6.9**	**86.1**	**9.3**	**0.001**
Ipsilateral lung volume (cc)	989.5	322	1154.2	498.4	0.06
**Ipsilateral lung-PTV volume (cc)**	**593.4**	**273.8**	**730.5**	**315.3**	**0.01**
**Ipsi lung-PTV mean dose (Gy)**	**34.0**	**4.5**	**31.8**	**4.4**	**0.002**
**Ipsi lung-PTV V20Gy (%)**	**86.7**	**10.6**	**77.2**	**15.3**	**<0.001**
Contralateral lung volume (cc)	1727	515	1692.3	377.5	0.68
Contra lung V5Gy (%)	49.5	13.0	48.2	23.5	0.72
Contra lung mean dose (Gy)	6.4	1.5	6.8	1.7	0.58
Contra lung V20Gy (%)	1.6	3.4	1.6	3.4	0.63
Total lung volume (cc)	2756	658	2851	667	0.39
Total lung Mean Dose (Gy)	18.3	2.9	19.2	1.7	0.13
Total lung V20Gy (%)	35.0	7.2	36.4	4.3	0.13
Total lung-PTV mean dose (Gy)	13.2	1.8	14.1	2.6	0.03
Total lung-PTV V20Gy (%)	22.3	7.2	25.4	4.9	0.07
Estimated NTCP mean (%)	9.7	2.4	9.1	1.6	0.20
Kidney					
Ipsi kidney Mean dose (Gy)	7.9	3.1	7.1	4.2	0.67
Ipsi kidney D2/3 (Gy)	3.4	1.3	4.0	3.4	0.31
**Contra kidney Mean dose (Gy)**	**2.7**	**1.9**	**3.7**	**1.8**	**0.02**
Contra kidney D2/3	1.4	1.1	2.2	2.1	0.11
Esophagus					
Mean dose (Gy)	22.2	10.0	25.9	9.6	0.20
**Max dose (Gy)**	**50.0**	**2.1**	**51.1**	**2.6**	**0.03**
Estimated NTCP mean (%)	2.4	3.1	3.1	3.3	0.37
Heart					
**Mean dose (Gy)**	**25.6**	**4.4**	**19.5**	**5.8**	**<0.001**
**V30Gy (%)**	**30.8**	**11.9**	**19.5**	**14.1**	**<0.001**
Spinal Cord					
Max dose (Gy)	38.0	8.5	39.1	6.3	0.16
Estimated NTCP mean (%)	0.01	0.04	0.03	0.05	0.11
Liver					
**Mean dose (Gy)**	**10.8**	**3.9**	**23.4**	**5.2**	**<0.001**
**V30Gy (%)**	**0.8**	**4.2**	**28.8**	**14.7**	**<0.001**
Stomach					
**Mean dose (Gy)**	**19.2**	**6.4**	**10.8**	**5.1**	**<0.001**
V45Gy (%)	7.4	7.7	4.4	3.0	0.06
**Estimated NTCP mean (%)**	**6.9**	**5.3**	**0.3**	**0.4**	**<0.001**

LSM *—left-sided mesothelioma; RSM *—right-sided mesothelioma; SD **—Standard deviation; IQR **—interquartile range; VXGy (%)—percentage of volume receiving X Gy; V100% (%)—percentage of PTV receiving 100% of the prescription dose; D2/3—dose delivered to 2/3 of volume; NTCP—Normal Tissue Complication Probability. The bold face indicates identified difference between LSM and RSM with *p* value < 0.05 level.

**Table 3:** Results of Cox PH regression based on dosimetric (right), clinical, and combined variables.

	HR	Lower CI	Upper CI	*p*-Value
Cox PH regression with dosimetric variables on the right side				
PTV_V100	0.77	0.42	1.42	0.4
PTV_Max	1.1	0.91	1.34	0.33
**PTV_Min**	**1.28**	**1.11**	**1.48**	**<0.001**
**Total_Lung_PTV_Mean**	**7.48**	**1.77**	**31.53**	**<0.01**
**Total_Lung_PTV_V20**	**0.53**	**0.33**	**0.85**	**<0.01**
Ipsi_Lung_PTV_Mean	0.85	0.63	1.14	0.28
**Contra_Lung_Volume**	**1**	**1**	**1**	**<0.01**
**Contra_Lung_V5**	**0.86**	**0.78**	**0.95**	**<0.01**
**Contra_Lung_V20**	**1.71**	**1.17**	**2.5**	**<0.01**
**Esophagus_Max**	**0.75**	**0.57**	**0.98**	**0.04**
**Esophagus_Mean**	**1.14**	**1.01**	**1.29**	**0.03**
**Heart_Volume**	**1.01**	**1**	**1.01**	**<0.01**
Heart_V5	1.88	0.96	3.68	0.06
(No stable fit with dosimetric variables on the left side was attained.) Cox PH regression with clinical variables				
**gender_male**	**4.23**	**1.22**	**14.63**	**0.02**
T Stage	0.66	0.41	1.07	0.09
**N Stage**	**1.60**	**1.07**	**2.38**	**0.02**
age	1.02	0.98	1.06	0.38
Cox PH regression with dosimetric and clinical variables				
Histology in Number (Epithelioid: 0; Mixed: 1)	1.25	0.49	3.22	0.64
gender_male	4.54	0.78	26.37	0.09
**T Stage**	**0.31**	**0.14**	**0.70**	**<0.01**
**N Stage**	**2.46**	**1.38**	**4.36**	**<0.01**
age	1.01	0.97	1.06	0.56
Pneumonitis	0.93	0.35	2.49	0.89
PTV_Side..L	5.32	0.87	32.57	0.07
PTV_Max	0.87	0.71	1.08	0.21
**PTV_Mean**	**1.67**	**1.01**	**2.76**	**<0.05**
**PTV_Min**	**1.12**	**1.01**	**1.24**	**0.04**
Total_Lung_PTV_Volume	1.00	1.00	1.00	0.51
Ipsi_Lung_PTV_Mean	0.93	0.78	1.11	0.42
Esophagus_Volume	1.01	0.97	1.06	0.59
Esophagus_Max	0.98	0.87	1.12	0.79
**Esophagus_Mean**	**1.14**	**1.03**	**1.27**	**0.01**
Heart_Volume	1.00	1.00	1.00	0.91
**Heart_V30**	**0.89**	**0.82**	**0.97**	**<0.01**
Spinal_Cord_Volume	1.02	0.99	1.04	0.23

The bold face indicates identified difference between LSM and RSM with *p* value < 0.05 level.

## Data Availability

The data presented in this study are available upon request from the corresponding authors.

## Supplementary Materials

The following supporting information can be downloaded at: https://www.mdpi.com/article/10.3390/cancers15153916/s1, Figure S1: Box plots of the selected dosimetric metrics between LSM and RSM groups. The dosimetric metrics include (a) PTV V100%, (b) PTV volume, (c) ipsilateral lung mean dose, (d) ipsilateral lung V20Gy, (e) ipsilateral lung - PTV mean dose, (f) contralateral kidney mean dose, (g) heart mean dose, (h) liver mean dose, (i) liver V30Gy, and (j) stomach mean dose; Table S1: potential dosimetric and clinical variables to be selected from; Table S2: summary of model performance for different settings. References [15,25,27,47,48] are cited in Supplementary file.
