# Supplementary material for: Predicting Overall Survival for Patients with Malignant Mesothelioma Following Radiotherapy via Interpretable Machine Learning
Source: Cancers (Basel). 2023 Aug 1;15(15):3916. doi: 10.3390/cancers15153916 (PMC10416916; doi:10.3390/cancers15153916)
Supplement: Supplementary file 1 [file cancers-15-03916-s001.zip › cancers-2485634-supplementary.pdf]

Supplementary: Statistical method

Recursive feature elimination (RFE)

As a well-known and commonly used feature selection algorithm, Recursive Feature Elimination (RFE), a. backward selection approach with the utilization of stepwise method [1-2], implements backwards selection of predictors according to predictor importance ranking. The survival model, as random survival forest (R package *mlr3extralearners*: lrn("surv.ranger")), with the right censored time was first trained on all potential predictors, the importance score was obtained via the permutation method. The predictor with lowest importance score was excluded. The model was refitted with the reduced pool of predictors as next step, following the model evaluation with 5-fold cross validation approach with C-index as performance measure. These steps were repeated iteratively until all features were traversed. This process enables the identification of optimal collection of predictors that this collection of predictors will be included into our final model [3].

Tuning process and hyperparameter of sSVM [4]

Two hyperparameters of sSVM, gamma and mu, was tuned with the following settings (R package *mlr3tuning*:AutoTuner()):

1. The hyperparameters of the wrapped (inner) learner are trained on the training data via resampling, which was specified as 5-folded cross-validation.
2. The measure to be optimized is set to be C-index.
3. Turner is set to apply the method of grid search.
4. Hyperparameter search space was set to be (0.001 and 1000) for both parameters gamma and mu.
5. The termination criteria was set to be after 4 evaluations

Supplementary Table S1: potential dosimetric and clinical variables to be selected from

	Dosimetric variables	Clinical variables
1	PTV_V100	Histology.in.Number..Epithelioid.0..Mixed..1.
2	PTV_Volume.cc.	gender_male
3	PTV_Max	T.Stage
4	PTV_Mean	N.Stage
5	PTV_Min	age
6	PTV.ipsi.PTV	Pneumonitis
7	Total_Lung_PTV_Volume	PTV_Side..L
8	Total_Lung_PTV_Mean	
9	Total_Lung_PTV_V20	
10	Ipsi_Lung_PTV_Volume	
11	Ipsi_Lung_PTV_Mean	

12	Ipsi_Lung_PTV_V20	
13	Contra_Lung_Volume	
14	Contra_Lung_Mean	
15	Contra_Lung_V5	
16	Contra_Lung_V20	
17	Esophagus_Volume	
18	Esophagus_Max	
19	Esophagus_Mean	
20	Heart_Volume	
21	Heart_Mean	
22	Heart_V5	
23	Heart_V30	
24	Heart_V50	
25	Spinal_Cord_Volume	
26	Spinal_Cord_Max	

Supplementary Table S2: summary of model performance for different settings

	Metrics of predictive performance**						
**Features**	**C-index (5-fold cross validation)**	van Houwelingen's Alpha Calibration	van Houwelingen's Beta Calibration	Integrated Graf Score	Integrated Log Loss	Log Loss	**Hyperparameter (tuned)**
Dosimetric variable – right							
Cox PH	0.54	0.69	0.02	0.27	1.10	34.54	
sSVM	0.56	NA	NA	NA	NA	NA	Gamma=1000, mu=0.001
Dosimetric variable – left							
Cox PH	0.47	Inf	-0.00	0.54	3.71	34.54	
sSVM	0.41	NA	NA	NA	NA	NA	Gamma=1000, mu=1000
Clinical variable							
Cox PH	0.70	1.12	3.29	0.21	0.62	31.39	
sSVM	0.65	NA	NA	N A	NA	NA	Gamma=0.001, mu=1000

Combined dosimetric and clinical variables							
Cox PH	0.62	0.36	0.26	0.29	1.14	31.18	
sSVM	0.64	NA	NA	NA	NA	NA	Gamma=0.001, mu=1000

*Gray indicates that stable fit was unattainable.

**the metrics of predictive performance are obtained via R package mlr3proba [5].

Supplemental Figure S1: Box plots of the selected dosimetric metrics between LSM and RSM groups. The dosimetric metrics include (a) PTV V100%, (b) PTV volume, (c) ipsilateral lung mean dose, (d) ipsilateral lung V20Gy, I ipsilateral lu–g - PTV mean dose, (f) contralateral kidney mean dose, (g) heart mean dose, (h) liver mean dose, (i) liver V30Gy, and (j) stomach mean dose.

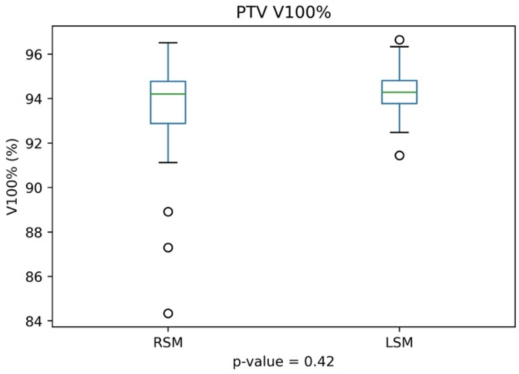

(a)

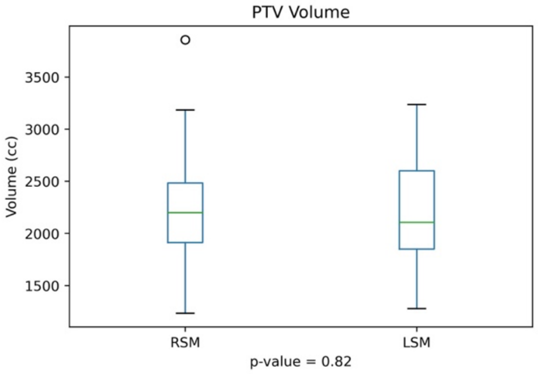

(b)

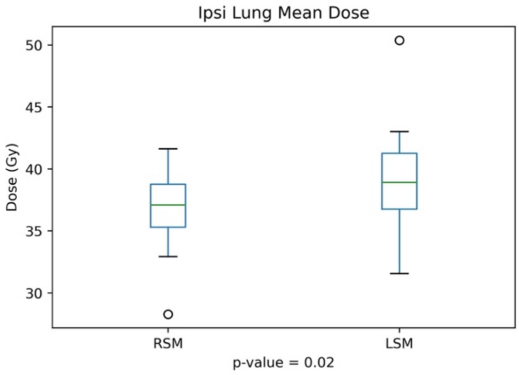

(c)

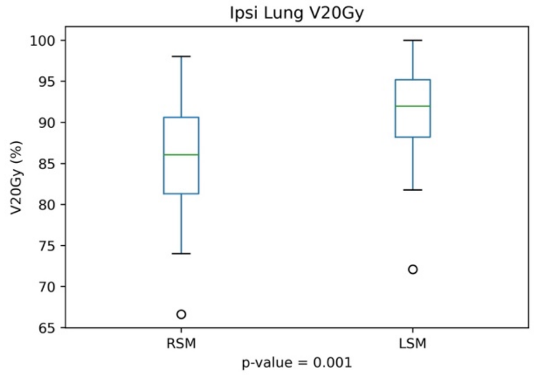

(d)

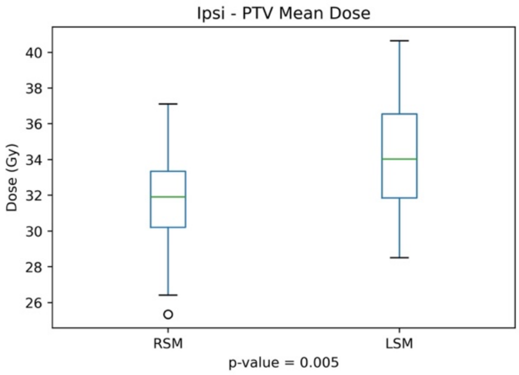

Ipsi - PTV Mean Dose
Dose (Gy)
40
38
36
34
32
30
28
26
RSM
LSM
p-value = 0.005

(e)

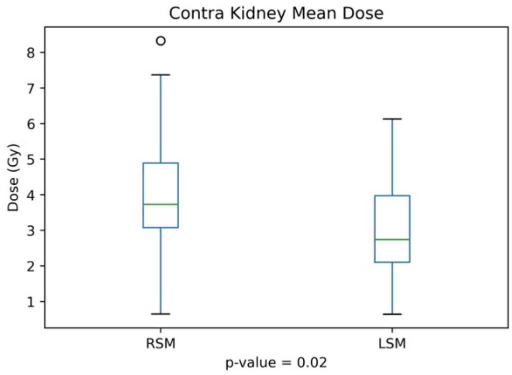

Contra Kidney Mean Dose
Dose (Gy)
8
7
6
5
4
3
2
1
RSM
LSM
p-value = 0.02

(f)

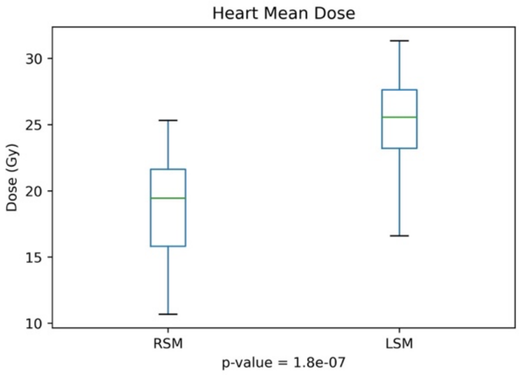

Heart Mean Dose
Dose (Gy)
30
25
20
15
10
RSM
LSM
p-value = 1.8e-07

(g)

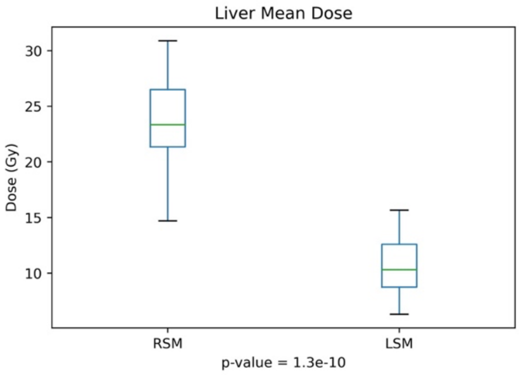

Liver Mean Dose
Dose (Gy)
30
25
20
15
10
RSM
LSM
p-value = 1.3e-10

(h)

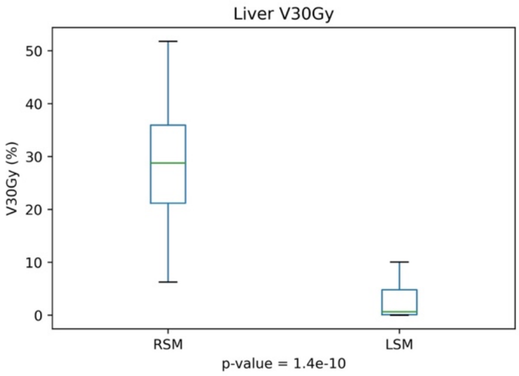

Liver V30Gy
V30Gy (%)
50
40
30
20
10
0
RSM
LSM
p-value = 1.4e-10

(i)

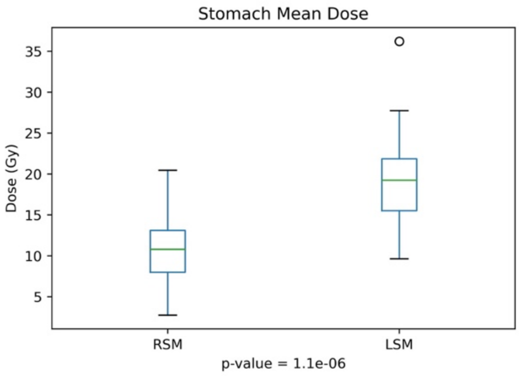

Stomach Mean Dose
Dose (Gy)
35
30
25
20
15
10
5
RSM
LSM
p-value = 1.1e-06

(j)

Reference:

1. Escanilla NS, Hellerstein L, Kleiman R, Kuang Z, Shull JD, Page D. Recursive Feature Elimination by Sensitivity Testing. Proc Int Conf Mach Learn Appl. 2018 Dec;2018:40-47. doi: 10.1109/ICMLA.2018.00014. Epub 2019 Jan 17. PMID: 31799516; PMCID: PMC6887481.
2. Giorgos Borboudakis and Ioannis Tsamardinos. 2019. Forward-backward selection with early dropping. J. Mach. Learn. Res. 20, 1 (January 2019), 276–314
3. Wu YL, Li WF, Yang KB, Chen L, Shi JR, Chen FP, Huang XD, Lin L, Zhang XM, Li J, Chen YP, Tang LL, Mao YP, Ma J. Long-Term Evaluation and Normal Tissue Complication Probability (NTCP) Models for Predicting Radiation-Induced Optic Neuropathy after Intensity-Modulated Radiation Therapy (IMRT) for Nasopharyngeal Carcinoma: A Large Retrospective Study in China. J Oncol. 2022 Feb 23;2022:3647462. doi: 10.1155/2022/3647462. PMID: 35251172; PMCID: PMC8890878.
4. Marc Becker, Michel Lang, Jakob Richter, Bernd Bischl and Daniel Schalk (2022). mlr3tuning: Tuning for 'mlr3'. R package version 0.15.0. https://CRAN.R-project.org/package=mlr3tuning
5. Sonabend R, Király FJ, Bender A, Bischl B, Lang M (2021). “mlr3proba: An R Package for Machine Learning in Survival Analysis.” Bioinformatics. ISSN 1367-4803, doi:10.1093/bioinformatics/btab039

Correlation Matrix of the Predictors

dose R

	PTV_V100	PTV_Max	PTV_Min	Total_Lung_	Total_Lung_	Ipsi_Lung_P	Contra_Lung	Contra_Lung	Contra_Lung	Esophagus_	Esophagus_	Heart_Volum	Heart_V5	Heart_V50
PTV_V100	1	-0.03	0.29	-0.03	0.01	0.51	0.03	-0.47	0.04	-0.15	-0.63	-0.15	-0.21	-0.04
PTV_Max	-0.03	1	-0.19	0.17	0.2	-0.19	-0.15	0.03	0.32	0.5	0.18	-0.11	0.22	0.13
PTV_Min	0.29	-0.19	1	-0.09	0.03	0.14	0.11	-0.15	-0.09	-0.17	-0.26	-0.1	-0.3	-0.27
Total_Lung_	-0.03	0.17	-0.09	1	0.85	0.12	0.1	0.37	0.17	0.16	-0.12	-0.28	0.31	-0.05
Total_Lung_	0.01	0.2	0.03	0.85	1	0.22	0.08	0.09	0.24	0.34	0.04	-0.25	0.19	-0.1
Ipsi_Lung_P	0.51	-0.19	0.14	0.12	0.22	1	0.02	-0.37	0.11	0.21	-0.08	0.04	-0.07	-0.02
Contra_Lung	0.03	-0.15	0.11	0.1	0.08	0.02	1	-0.02	-0.04	0.04	-0.1	-0.19	-0.14	0.07
Contra_Lung	-0.47	0.03	-0.15	0.37	0.09	-0.37	-0.02	1	0.35	-0.33	0.18	-0.07	0.51	-0.02
Contra_Lung	0.04	0.32	-0.09	0.17	0.24	0.11	-0.04	0.35	1	0.32	0.11	-0.36	0.19	-0.06
Esophagus_	-0.15	0.5	-0.17	0.16	0.34	0.21	0.04	-0.33	0.32	1	0.25	-0.12	0.02	0.19
Esophagus_	-0.63	0.18	-0.26	-0.12	0.04	-0.08	-0.1	0.18	0.11	0.25	1	0.01	0.16	0.12
Heart_Volum	-0.15	-0.11	-0.1	-0.28	-0.25	0.04	-0.19	-0.07	-0.36	-0.12	0.01	1	-0.1	0.14
Heart_V5	-0.21	0.22	-0.3	0.31	0.19	-0.07	-0.14	0.51	0.19	0.02	0.16	-0.1	1	0.2
Heart_V50	-0.04	0.13	-0.27	-0.05	-0.1	-0.02	0.07	-0.02	-0.06	0.19	0.12	0.14	0.2	1

dose L

	PTV_Volume	PTV_Max	PTV_Mean	PTV.ipsi.PTV	Total_Lung_	Ipsi_Lung_P	Ipsi_Lung_P	Contra_Lung	Contra_Lung	Esophagus_	Esophagus_	Esophagus_	Heart_Mean	Heart_V5	Heart_V50	Spinal_Cord_Volume
PTV_Volume	1	0.07	-0.01	0.27	0.07	-0.33	0	0.34	0.22	0.2	0.15	0.04	0.21	-0.13	0.17	0.47
PTV_Max	0.07	1	0.71	-0.07	0.16	-0.14	-0.24	0.37	0.22	-0.06	0.42	-0.21	-0.1	0.03	0.18	0.01
PTV_Mean	-0.01	0.71	1	0.12	0.15	0.1	0.09	0.48	0.23	-0.2	0.26	0.26	0.34	0.26	0.6	0.04
PTV.ipsi.PTV	0.27	-0.07	0.12	1	-0.71	0.45	0.61	-0.11	-0.2	-0.18	-0.11	-0.07	0.01	0.16	0.02	-0.1
Total_Lung_	0.07	0.16	0.15	-0.71	1	-0.46	-0.57	0.57	0.46	0.24	0.1	0.21	0.24	0.06	0.39	0.22
Ipsi_Lung_P	-0.33	-0.14	0.1	0.45	-0.46	1	0.81	-0.38	-0.52	-0.13	-0.43	-0.06	0.07	-0.02	-0.07	-0.16
Ipsi_Lung_P	0	-0.24	0.09	0.61	-0.57	0.81	1	-0.28	-0.23	-0.08	-0.49	0.06	0.29	0.13	-0.01	-0.04
Contra_Lung	0.34	0.37	0.48	-0.11	0.57	-0.38	-0.28	1	0.66	0.2	0.24	0.27	0.42	0.23	0.61	0.02
Contra_Lung	0.22	0.22	0.23	-0.2	0.46	-0.52	-0.23	0.66	1	0.41	0.1	0.19	0.27	0.31	0.17	0.09
Esophagus_	0.2	-0.06	-0.2	-0.18	0.24	-0.13	-0.08	0.2	0.41	1	-0.12	0.24	0.03	0.1	-0.05	0.34
Esophagus_	0.15	0.42	0.26	-0.11	0.1	-0.43	-0.49	0.24	0.1	-0.12	1	0.19	0.09	0.04	0.1	-0.12
Esophagus_	0.04	-0.21	0.26	-0.07	0.21	-0.06	0.06	0.27	0.19	0.24	0.19	1	0.71	0.43	0.46	0.17
Heart_Mean	0.21	-0.1	0.34	0.01	0.24	0.07	0.29	0.42	0.27	0.03	0.09	0.71	1	0.52	0.49	0.03
Heart_V5	-0.13	0.03	0.26	0.16	0.06	-0.02	0.13	0.23	0.31	0.1	0.04	0.43	0.52	1	0.22	-0.11
Heart_V50	0.17	0.18	0.6	0.02	0.39	-0.07	-0.01	0.61	0.17	-0.05	0.1	0.46	0.49	0.22	1	0.21
Spinal_Cord	0.47	0.01	0.04	-0.1	0.22	-0.16	-0.04	0.02	0.09	0.34	-0.12	0.17	0.03	-0.11	0.21	1

clinical

	gender_male	T.Stage	N.Stage	age
gender_male	1	0.11	0.03	0.24
T.Stage	0.11	1	0.36	-0.09
N.Stage	0.03	0.36	1	-0.23
age	0.24	-0.09	-0.23	1

clinical dose

	Histology.in	gender_male	T.Stage	N.Stage	age	cond	PTV_Side..L.	PTV_Max	PTV_Mean	PTV_Min	Total_Lung_	Ipsi_Lung_P	Esophagus_	Esophagus_	Esophagus_	Heart_Volum	Heart_V30	Spinal_Cord_Volume
Histology.in	1	0.14	-0.26	-0.08	0.14	0.04	0.07	0.08	-0.08	-0.03	-0.1	0.03	0.18	0.1	0.17	-0.01	0.13	-0.18
gender_male	0.14	1	0.11	0.03	0.24	0.08	-0.2	0.17	0.01	0.11	0.43	-0.31	0.32	-0.01	-0.16	0.38	-0.26	0.12
T.Stage	-0.26	0.11	1	0.36	-0.09	0.01	-0.08	0.28	0.37	-0.15	-0.08	-0.26	0.12	0.03	-0.04	0.02	-0.19	-0.04
N.Stage	-0.08	0.03	0.36	1	-0.23	0.12	-0.12	0.21	0.28	0.07	-0.17	0.03	-0.11	0.18	0.14	0.11	0.17	-0.25
age	0.14	0.24	-0.09	-0.23	1	-0.11	-0.18	-0.17	-0.17	0.07	0.07	-0.19	0.25	-0.17	0.03	0.16	-0.13	-0.01
cond	0.04	0.08	0.01	0.12	-0.11	1	-0.25	0.06	-0.19	0.06	-0.25	-0.3	0.01	0.04	0.18	0.03	-0.09	0.07
PTV_Side..L.	0.07	-0.2	-0.08	-0.12	-0.18	-0.25	1	-0.12	-0.03	0.04	-0.11	0.44	-0.21	-0.1	-0.09	0	0.59	0.04
PTV_Max	0.08	0.17	0.28	0.21	-0.17	0.06	-0.12	1	0.59	-0.16	0.04	-0.21	0.04	0.47	0.05	-0.08	-0.11	-0.21
PTV_Mean	-0.08	0.01	0.37	0.28	-0.17	-0.19	-0.03	0.59	1	-0.28	-0.04	0.06	0.03	0.38	0.05	0.04	0.12	-0.11
PTV_Min	-0.03	0.11	-0.15	0.07	0.07	0.06	0.04	-0.16	-0.28	1	0.15	0.03	-0.13	-0.15	-0.18	0.08	-0.03	0.05
Total_Lung_	-0.1	0.43	-0.08	-0.17	0.07	-0.25	-0.11	0.04	-0.04	0.15	1	-0.22	0.16	0.07	-0.15	-0.07	-0.15	0.18
Ipsi_Lung_P	0.03	-0.31	-0.26	0.03	-0.19	-0.3	0.44	-0.21	0.06	0.03	-0.22	1	-0.21	-0.01	-0.1	0.16	0.27	-0.13
Esophagus_	0.18	0.32	0.12	-0.11	0.25	0.01	-0.21	0.04	0.03	-0.13	0.16	-0.21	1	0.11	0.19	0.27	-0.02	-0.01
Esophagus_	0.1	-0.01	0.03	0.18	-0.17	0.04	-0.1	0.47	0.38	-0.15	0.07	-0.01	0.11	1	0.24	-0.18	0.19	-0.21
Esophagus_	0.17	-0.16	-0.04	0.14	0.03	0.18	-0.09	0.05	0.05	-0.18	-0.15	-0.1	0.19	0.24	1	-0.1	0.46	0.08
Heart_Volum	-0.01	0.38	0.02	0.11	0.16	0.03	0	-0.08	0.04	0.08	-0.07	0.16	0.27	-0.18	-0.1	1	0.01	-0.14
Heart_V30	0.13	-0.26	-0.19	0.17	-0.13	-0.09	0.59	-0.11	0.12	-0.03	-0.15	0.27	-0.02	0.19	0.46	0.01	1	-0.11
Spinal_Cord	-0.18	0.12	-0.04	-0.25	-0.01	0.07	0.04	-0.21	-0.11	0.05	0.18	-0.13	-0.01	-0.21	0.08	-0.14	-0.11	1
